# Human Muse cells isolated from preterm- and term-umbilical cord delivered therapeutic effects in rat bleomycin-induced lung injury model without immunosuppressant

**DOI:** 10.1186/s13287-024-03763-8

**Published:** 2024-05-22

**Authors:** Kaung Htet Nay Win, Yoshihiro Kushida, Keiji Yamana, Sota Iwatani, Makiko Yoshida, Nanako Nino, Cho Yee Mon, Hiroyuki Ohsaki, Shingo Kamoshida, Kazumichi Fujioka, Mari Dezawa, Noriyuki Nishimura

**Affiliations:** 1 Department of Public Health, Kobe University Graduate School of Health Science, 7-10-2 Tomogaoka, Suma-ku, Kobe, Hyogo 654-0142 Japan; 2 Department of Stem Cell Biology and Histology, Tohoku University Graduate School of Medicine, 2-1, Seiryo-Machi, Aoba-ku, Sendai, Miyagi 980-8575 Japan; 3 Department of Pediatrics, Kobe University Graduate School of Medicine, Kobe, Hyogo Japan; 4 Department of Neonatology, Kobe Children’s Hospital, Kobe, Hyogo Japan; 5 Department of Pathology, Kobe Children’s Hospital, Kobe, Hyogo Japan; 6 Department of Medical Biophysics, Kobe University Graduate School of Health Science, Kobe, Hyogo Japan

**Keywords:** Preterm UC-Muse cells, Term UC-Muse cells, BM-Muse cells, BLM-induced lung injury, BPD, IPF, COPD

## Abstract

**Background:**

Bleomycin (BLM)-induced lung injury is characterized by mixed histopathologic changes with inflammation and fibrosis, such as observed in human patients with bronchopulmonary dysplasia, idiopathic pulmonary fibrosis, and chronic obstructive pulmonary disease. Although no curative therapies for these lung diseases exist, stem cell therapy has emerged as a potential therapeutic option. Multilineage-differentiating stress-enduring (Muse) cells are endogenous pluripotent- and macrophage-like stem cells distributed in various adult and fetal tissues as stage-specific embryonic antigen-3-positive cells. They selectively home to damaged tissue by sensing sphingosine-1-phosphate and replace the damaged/apoptotic cells by in vivo differentiation. Clinical trials for some human diseases suggest the safety and therapeutic efficacy of intravenously injected human leukocyte antigen-mismatched allogenic Muse cells from adult bone marrow (BM) without immunosuppressant. Here, we evaluated the therapeutic effects of human Muse cells from preterm and term umbilical cord (UC), and adult BM in a rat BLM-induced lung injury model.

**Methods:**

Rats were endotracheally administered BLM to induce lung injury on day 0. On day 3, human preterm UC-Muse, term UC-Muse, or adult BM-Muse cells were administered intravenously without immunosuppressants, and rats were subjected to histopathologic analysis on day 21. Body weight, serum surfactant protein D (SP-D) levels, and oxygen saturation (SpO_2_) were monitored. Histopathologic lung injury scoring by the Ashcroft and modified American Thoracic Society document scales, quantitative characterization of engrafted Muse cells, RNA sequencing analysis, and in vitro migration assay of infused Muse cells were performed.

**Results:**

Rats administered preterm- and term-UC-Muse cells exhibited a significantly better recovery based on weight loss, serum SP-D levels, SpO_2_, and histopathologic lung injury scores, and a significantly higher rate of both Muse cell homing to the lung and alveolar marker expression (podoplanin and prosurfactant protein-C) than rats administered BM-Muse cells. Rats receiving preterm-UC-Muse cells showed statistically superior results to those receiving term-UC-Muse cells in many of the measures. These findings are thought to be due to higher expression of genes related to cell migration, lung differentiation, and cell adhesion.

**Conclusion:**

Preterm UC-Muse cells deliver more efficient therapeutic effects than term UC- and BM-Muse cells for treating BLM-induced lung injury in a rat model.

**Supplementary Information:**

The online version contains supplementary material available at 10.1186/s13287-024-03763-8.

## Introduction

Bleomycin (BLM) is a widely used reagent for inducing lung injury in animal models that exhibit histopathologic lung changes characterized by inflammation and fibrosis [1]. BLM-induced lung injury is mediated by DNA strand breaks through the production of DNA-cleaving superoxide and hydroxyl free radicals [2], as well as by the low expression of BLM-inactivating enzyme (BLM-hydrolase) [3], leading to patchy parenchymal inflammation, fibroblast activation, extracellular matrix deposition, and alterations of smooth muscle and microvasculature in the lung [4]. These structural changes are also observed in human patients with preterm infant-related lung complications of bronchopulmonary dysplasia (BPD) [5, 6] and age-related lung diseases of idiopathic pulmonary fibrosis (IPF) [4] and chronic obstructive pulmonary disease (COPD) [7].

BPD was first described in 1967 as a chronic lung injury in preterm infants resulting from supplemental oxygen and mechanical ventilation [8] and is currently the most frequent complication of preterm infants associated with a higher risk of various long-term lung complications and adverse neurodevelopmental outcomes [9]. Preterm infants usually suffer from lung injury requiring supplemental oxygen and mechanical ventilation, and eventually manifest BPD. The pathology of BPD results from lung injury and abnormal lung repair, and is characterized by impaired alveolar and vascular development of the immature lung. In addition to supplemental oxygen and mechanical ventilation, inflammation, oxidative stress, and exhaustion and/or dysfunction of resident lung stem cells are implicated as the main causes of BPD [10, 11].

No curative therapies exist for these lung diseases, but stem cell therapy has emerged as a potential therapeutic option. Mesenchymal stem cells (MSCs) were first isolated from the bone marrow (BM) [12, 13] and later from a variety of tissues such as the adipose tissue, dermis, amnion, and umbilical cord (UC) [14]. The therapeutic effects of these types of MSCs have been evaluated in various lung diseases. Although no currently available animal models faithfully reproduce all the features of human lung diseases [15], MSCs migrate to the damaged lung after intravenous injection, exert bystander effects such as anti-inflammation and anti-fibrosis effects, and deliver therapeutic results in animal models of BLM-induced lung injury [16]. In human clinical trials, however, the therapeutic effects of MSCs on BPD, IPF, and COPD are limited due to their transient survival in the injured lung and their inability to differentiate into the lung components to replace damaged cells [17–20].

Multilineage-differentiating stress-enduring (Muse) cells are endogenous pluripotent-like/macrophage-like stem cells distributed in the BM, peripheral blood, and organ connective tissues as stage-specific embryonic antigen (SSEA)-3-positive cells [21–23]. They exhibit low telomerase activity and are non-tumorigenic [24]. In addition to biologic tissues, Muse cells can be obtained as several percent of MSCs, and their unique characteristics make them distinct from the other cells in MSCs, namely non-Muse MSCs [21, 24, 25]. Muse cells express sphingosine-1-phosphate (S1P) receptor 2 that senses the damage signal S1P produced by damaged cells, and selectively migrate to and home to the damaged tissue rather than being trapped in the lung capillaries [26]. After homing to the damaged tissue, Muse cells phagocytose damaged/apoptotic cells, directly recycle signals from the up-taken damaged/apoptotic cells necessary for differentiation, such as transcription factors, and quickly differentiate into the same cell type as the phagocytosed damaged/apoptotic cells [23]. This type of differentiation proceeds rapidly compared with in vitro cytokine-induced differentiation; cytokine induction into melanocytes, cardiomyocytes, and neural-lineage cells takes at least several weeks to months for ~ 80% of Muse cells to differentiate into target cell types, while the phagocytosis-induced differentiation proceeds within days [23, 27–29]. In this manner, they replace various types of damaged/apoptotic cells with healthy functioning cells in the damaged tissue because they are pluripotent-like [23].

Notably, human leukocyte antigen (HLA)-mismatched donor-Muse cells can be directly administered to patients without immunosuppressant treatment due to their specific immunotolerance, partially explained by the expression of HLA-G, relevant to the immunotolerance in the placenta during pregnancy [26, 30]. Based on these beneficial properties, intravenously administered allogenic-Muse cells have been tested in clinical trials for acute myocardial infarction [31], stroke [32], epidermolysis bullosa [33], amyotrophic lateral sclerosis [34], spinal cord injury, neonatal hypoxic-ischemic encephalopathy, and acute respiratory distress syndrome, all without HLA matching test or immunosuppressant treatment, and safety and efficacy of Muse cells are suggested [31–34].

Muse cells derived from different sources such as BM, adipose tissue, dermis, and amnion consistently exhibit pluripotency gene expression, trilineage-differentiation ability, and self-renewal at a single-cell level [21, 35–37]. The differentiation tendencies differ among Muse cells [21, 35]: Adipose tissue-Muse cells and dermis-Muse cells express higher levels of mesodermal- and ectodermal-lineage markers than BM-Muse cells, respectively, while amnion-Muse cells show higher potential to differentiate into germ-line and extraembryonic-lineage cells compared with BM-Muse cells [36]. While Muse cells have also been harvested from UC [38] and preterm UC yields more vigorously proliferative Muse-cell source MSCs with a higher differentiation capacity than term UC [38–40], the basic characteristics and therapeutic effects of preterm- and term-UC-Muse cells have not been investigated.

In the present study, we evaluated the therapeutic effects of the 3 kinds of human Muse cells obtained from preterm-UC, term-UC, and adult BM on ameliorating rat BLM-induced lung injury model without immunosuppression. We also compared the characteristics of the 3 types of Muse cells in terms of their gene expression patterns and in vitro migration activities to the injured lung tissue.

## Methods

### Human UC samples

Human UCs were obtained from 6 infants delivered either at 23 weeks (preterm, 1 male and 2 females) or 38 weeks (term, 3 females) of gestation following written parental consent (Table S1). The present study was approved by the Ethics Committee at Kobe University Graduate School of Medicine (No.1370 and No.1431) and Hyogo Prefectural Kobe Children’s Hospital (No. 24–25) and conducted according to the Guidelines for the Clinical Research of Kobe University Graduate School of Medicine. All patients or their guardians provided written informed consent for the use of UC samples.

### Preparation of human UC- and BM-MSCs

Human preterm- and term-UC-MSCs were isolated and cultured as described previously [39, 41, 42]. Briefly, preterm- and term-UC (2–3 g wet weight) were collected, cut into 2–3 mm^3^ pieces, enzymatically dissociated with Liberase DH Research Grade (Roche, Mannheim, Germany), and filtered through a 100 µm cell strainer (BD Bioscience, Bedford, MA, USA). Collected cells were cultured at 37 °C (5% CO_2_ and 95% air) in alpha-minimum essential medium (Wako Pure Chemical, Osaka, Japan) containing 10% fetal bovine serum (Millipore Sigma, St. Louis, MO, USA) and 1% antibiotic–antimycotic solution (Invitrogen, Carlsbad, CA) until reaching confluency and then subcultured.

Human BM-MSCs purchased from Lonza (Tokyo, Japan) were cultured at 37°C (5% CO_2_ and 95% air) in low-glucose Dulbecco’s modified Eagle medium (Life Technologies, Carlsbad, CA, USA) containing 10% fetal bovine serum and 0.1 mg/mL kanamycin (Invitrogen) until reaching confluency and were subcultured as described previously [43].

### Preparation of human Muse and non-Muse cells

Human MSCs (fifth to eighth passages) were labeled with green fluorescent protein (GFP)-lentivirus as described previously [44, 45]. GFP(+)/SSEA-3(+) and GFP(+)/SSEA-3(−) cells were collected from GFP-labeled-MSCs as Muse and non-Muse cells, respectively, using a fluorescence-activated cell sorter (FACSAria II, Becton Dickson, Franklin Lakes, NJ) [46]. Human preterm UC-Muse, term UC-Muse, BM-Muse, and non-Muse cells were prepared from preterm UC-MSCs, term UC-MSCs, BM-MSCs, and BM-MSCs, respectively. In the present study, all human Muse cells were used at passages 6 to 9.

### BLM-induced rat lung injury model

All animal care and experiments were approved by the Committee of Animal Experiments at the Kobe University Graduate School of Medicine (No. P140703). Animal experiments were conducted according to the Guidelines for the Care and Use of Laboratory Animals of Kobe University Graduate School of Medicine and were reported in line with the ARRIVE guidelines 2.0. Male 6-week-old Lewis rats were obtained from SLC (Shizuoka, Japan) and maintained under standard conditions with free access to water and laboratory rodent food. At the start of the study (day 0), Lewis rats weighing 180 to 200 g were anesthetized with isoflurane (Wako Pure Chemical) in a closed box. Anesthetized rats were endotracheally intubated with aerosol sprayers (Natsume Seisakusho, Tokyo, Japan). They were given a single dose of BLM (Nippon Kayaku, Tokyo, Japan, 12 mg/kg in 300 μl dH_2_O) or the same volume of phosphate-buffered saline (PBS). On day 3, preterm UC-Muse cells, term UC-Muse cells, BM-Muse cells, and BM-non-Muse cells (non-Muse cells), all at 1 × 10^5^ cells/1.0 ml PBS, or the same volume of PBS were injected into the tail vein without immunosuppressant treatment. On day 21, all rats were killed under deep anesthesia with an overdose of isoflurane and subjected to histopathologic analysis. Body weight was monitored and recorded on days 0, 1, 2, 3, 4, 5, 7, 10, 14, 17, and 21.

### Surfactant protein D (SP-D) measurements

The peripheral blood was sampled from the tail vein without anesthesia on day 14, and the serum was separated and stored at − 80 °C until use. SP-D levels were measured using a Rat/Mouse SP-D ELISA kit (Yamasa, Tokyo, Japan) according to the manufacturer’s instructions.

### Cardiorespiratory assessment

Cardiorespiratory assessment was performed using a mouse/rat pulse oximeter MouseOX Plus (Starr Life Sciences, Oakmont, PA, USA) in freely moving rats that wore a neck collar sensor to detect vital signs from the carotid artery. On day 7, the fur around the neck area was removed under isoflurane anesthesia. On day 8, the rats were allowed to acclimatize to the sensor for 2 h to minimize the potential stress during the cardiorespiratory assesment. On day 9, oxygen saturation (SpO_2_) and heart rate were recorded for 60 min.

### Preparation of tissue sections

On day 21, all rats were killed by an overdose of isoflurane and cervical dislocation, and the lung, heart, intestine, kidney, spleen, and liver were dissected out. For paraffin sections, the right and left lungs were inflated with a 10% buffered neutral formalin solution (Muto Pure Chemicals, Tokyo, Japan) through the trachea before fixation and all tissues were fixed for 24 h in a 10% buffered neutral formalin solution (Muto Pure Chemicals) at room temperature, embedded in paraffin, and cut into 4-μm-thick sections. For cryosections, the right and left lungs were inflated with 4% paraformaldehyde (PFA) in 0.1 M PBS through the trachea and fixed for 24 h in 4% PFA in 0.1 M PBS at 4 °C, embedded in Optimal Cutting Temperature (OCT) compound (Sakura Fine Technical Co., Ltd., Tokyo, Japan), and then cut into 6 µm-thick frozen sections.

### Histopathologic assessments

Paraffin sections were deparaffinized in xylene, rehydrated in alcohol, and stained with hematoxylin and eosin (H&E). To evaluate the severity of lung injury on day 21, H&E-stained sections were analyzed and scored according to the Ashcroft scale [47, 48] and the modified American Thoracic Society (ATS) document scale [49, 50]. In the Ashcroft scale, lung fibrosis was scored from 0 to 8 in 20 randomly selected fields by a pathologist blind to the treatment groups [47, 48]. In the modified ATS document scale, 8 pathologic categories (area of aberration, granuloma, necrosis, cyst, alveolar septal thickness, neutrophils, lymphocytes, and nuclear debris) were scored from 0 to 23 in 45 randomly selected fields by a pathologist blinded to the treatment groups [49, 50].

### Immunohistochemistry

To detect the injected human Muse cells, paraffin sections were deparaffinized in xylene, rehydrated in alcohol, blocked in 3% H_2_O_2_ and Blocking One (Nacalai Tesque, Kyoto, Japan), incubated with anti-GFP rabbit polyclonal antibody (MBL, Nagoya, Japan) followed by horseradish peroxidase-conjugated anti-rabbit IgG goat polyclonal antibody (dilution 1:1,00, Nichirei Bioscience, Tokyo, Japan), visualized by 3, 3′-diaminobenzidine tetra-hydrochloride, and counterstained with hematoxylin. Images were captured using a BX53 microscope (Evident, Tokyo, Japan). For counting GFP-positive cells, 15 randomly captured images per group (3 non-overlapping random visual fields per paraffin section; n = 5/group) were subjected to BZ-X800 Analyzer software (Keyence, Osaka, Japan).

To evaluate the differentiation of injected cells in the rat lung, frozen sections were washed with PBS, incubated with 20% Block-Ace/5% bovine serum albumin/0.3% Triton X-100 in PBS solution for blocking, incubated with rabbit anti-GFP antibody (1:100; abcam, ab6556) or goat anti-GFP antibody (1:200; abcam, ab6673), followed by incubation with secondary antibody of either Alexa Fluor 488-conjugated donkey anti-rabbit IgG antibody (1:200: Jackson ImmunoResearch, West Grove, PA, USA; 711-546-152) or Alexa Fluor 488-conjugated donkey anti-goat IgG antibody (1:200: Jackson ImmunoResearch, 705-545-003). After washing with PBS, the sections were incubated with either mouse anti-podoplanin (a marker for type I alveolar epithelial cells; 1:200; abcam, ab10288), rabbit anti-prosurfactant protein C (proSP-C; a marker for type II alveolar epithelial cells; 1:100; MilliporeSigma, AB3786), or goat anti-CD31 antibodies (a marker for endothelial cells; 1:50; Santa Cruz Biotechnology, Dallas, TX, USA, sc-1506), followed by incubation with secondary antibody of either Alexa Fluor 594-conjugated donkey anti-mouse IgG antibody (1:200: Jackson ImmunoResearch, 715-586-150), Alexa Fluor 594-conjugated donkey anti-rabbit IgG antibody (1:200: Jackson ImmunoResearch, 711-586-152), or Alexa Fluor 594-conjugated donkey anti-goat IgG antibody (1:200: Jackson ImmunoResearch, 705-586-147). The sections were counterstained by 4′,6-diamidino-2-phenylindole (DAPI; 1:500; Thermo Fisher Scientific, D1306). Images were acquired with a confocal laser microscope (A1; Nikon). Cells double positive for GFP, and either podoplanin, pro-SPC, or CD31 were quantified using ImageJ.1.53t and calculated from 5 randomly obtained images in each section [51].

### RNA sequencing analysis

Total RNA was isolated using the NucleoSpin RNA (Macherey–Nagel, Duren, Germany, 740,902.50). The extracted RNA’s purity and concentration were evaluated using an Agilent 2100 bioanalyzer (Agilent Technologies, Palo Alto, CA, USA). The library was constructed by NEB Next Poly(A) mRNA Magnetic Isolation Module (New England Biolabs, Hitchin, UK, E7490) and NEB Next Ultra RNA Library Prep Kit for Illumina (NEB, E7530). Sequencing was performed on a NovaSeq 6000 (Illumina) by Rhelixa (Tokyo, Japan). Quality control and adapter trimming of sequencing data were performed using fastp version 0.20.0 [52]. DNA reads were mapped to the human reference genome DNA (build GRCh38.94) by HISAT2 version 2.2.1 with the default setting [53]. Gene counts from the mapped HISAT2 output for downstream expression analysis were generated by featureCounts version 2.0.1 [54]. Read counts for each gene were normalized to transcripts per million. Hierarchical clustering with heatmap was generated using iDEP version 0.96 [55]. To compare the biologic processes of preterm UC-Muse, term UC-Muse, and BM-Muse cells, Metascape was used for the pathway and process enrichment analysis with default parameters [56]. For marker genes of the lung and blood vessel development and cell migration, we selected genes annotated with gene ontology terms related to “lung development,” “blood vessel development,” “cell migration,” and “cell adhesion.”

### Migration assay

The experiment was performed according to the manufacturer’s protocol using a Matrigel invasion chamber (BD, 354,480). Serum-free medium alone or lung tissue slices obtained from intact Wistar rats in alpha-minimum essential medium were placed in the lower chamber. Human preterm UC-Muse, term UC-Muse, BM-Muse, or BM-non-Muse cells (2.5 × 10^4^) were placed onto the upper chamber and incubated at 37 °C, 5% CO_2_ for 24 h. Migrated cells were fixed with 4% PFA in 0.1 M phosphate buffer for 15 min and stained with Mayer’s hematoxylin (Fujifilm, Tokyo, Japan, 131-09665). The number of migrated cells was counted under 20 × objectives in 4 fields for each sample, and the mean of 3 samples was calculated using ImageJ.1.53t.

### Statistical analysis

All data are shown as mean ± standard error of the mean (SEM). Group comparisons were performed using a 1-way ANOVA followed by the Holm-Sidak multiple comparisons test for more than 2 groups and Student’s t test for 2 groups. A *p* value less than 0.05 was considered statistically significant. GraphPad Prism (version 10.2, GraphPad Software, Boston, MA) was used for statistical analyses.

## Results

### Body weight in the BLM-induced lung injury model

**Fig. 1:**
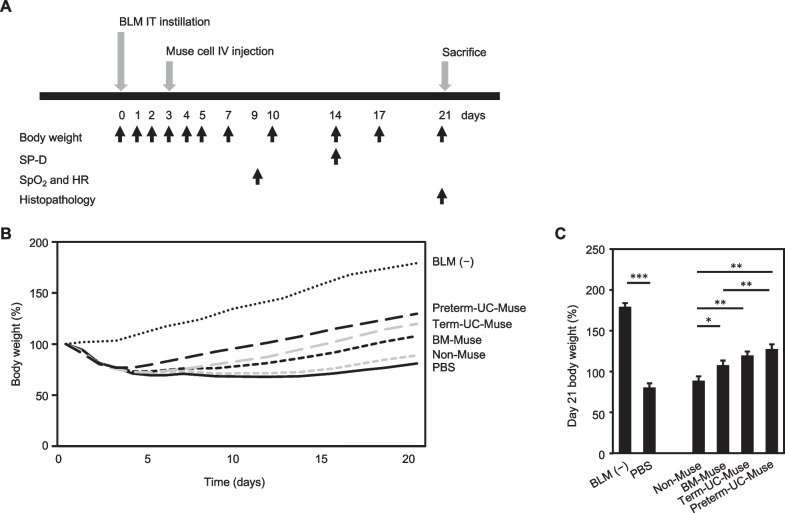
Body weight changes in BLM-induced lung injury model rats. **A** Schema of experimental design. BLM, bleomycin; IT, intratracheal; IV, intravenous; SpO_2_, oxygen saturation; HR, heart rate. **B** Body weight changes measured at days 0, 1, 2, 3, 4, 5, 7, 10, 14, 17, and 21 (n = 5/group). The value at day 0 was set to 100%. BLM(−) = non-injured intact group; PBS = BLM-injured PBS-treated group; Non-Muse = BLM-injured non-Muse cell-treated group; BM-Muse = BLM-injured BM-Muse cell-treated group; Term UC-Muse = BLM-injured term UC-Muse cell-treated group; Preterm UC-Muse = BLM-injured preterm UC-Muse cell-treated group. **C** Body weight at day 21. *p < 0.05, **p < 0.01, and ***p < 0.001

To evaluate the therapeutic effects of UC-Muse cells on BLM-induced lung injury,

male Lewis rats weighing 180–200 g were intratracheally (IT) administered BLM (12 mg/kg) on day 0. They received either 1 × 10^5^ preterm-UC-Muse, term-UC-Muse, BM-Muse, or non-Muse cells by intravenous injection on day 3 and were killed on day 21 (Fig. 1A). Control rats received the same volume of PBS intravenously. All the groups except the BLM(−) intact group showed body weight loss until 3 days after BLM administration. Unlike the PBS group, the preterm-UC-Muse, term-UC-Muse, BM-Muse groups exhibited recovery after 5 days (Fig. 1B). The non-Muse group showed marginal recovery compared with the 3 Muse groups (Fig. 1B). At day 21, the preterm-UC-Muse group had the highest recovery of body weight among the groups; the body weight was significantly higher than that of the BM-Muse (*p* < 0.01) and non-Muse groups (*p* < 0.01), but not the term-UC-Muse group (*p* = 0.370; Fig. 1C). The non-Muse group exhibited the lowest body weight recovery at day 21; the mean body weight did not differ significantly from that in the PBS group (*p* = 0.351; Fig. 1C). The body weights of the term-UC-Muse and BM-Muse groups did not differ significantly from each other at day 21 (*p* = 0.335; Fig. 1C).

### Serum SP-D level

**Fig. 2:**
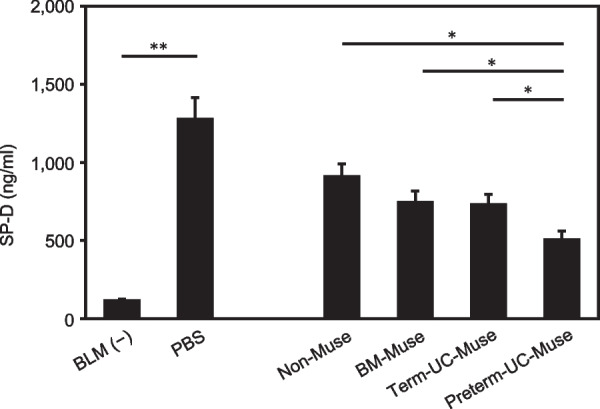
Serum SP-D levels at day 14. Five animals were used for each group. *p < 0.05 and **p < 0.01

As SP-D more closely correlates with the severity of BLM-induced lung injury than other conventional biomarkers such as lactate dehydrogenase, monocyte chemoattractant protein-1, aspartate aminotransferase, alanine aminotransferase, and high mobility group box l, and C-reactive protein [57], the SP-D serum level was measured at day 14 (Fig. 1A). The SP-D levels were significantly higher in the PBS group (1285.2 ± 129.6 ng/mL) than in the BLM(-) intact group (122.2 ± 4.2 ng/mL; *p* < 0.01) and decreased following Muse cell treatment, suggesting that BLM administration induced lung injury. SP-D in the preterm-UC-Muse group was the lowest among the treated groups and the difference was significantly different from that in the term-UC-Muse, BM-Muse, and non-Muse groups (all with *p* < 0.05). In contrast, the differences in the SP-D levels were not significant among the term-UC-Muse, BM-Muse, and non-Muse groups (Fig. 2), suggesting that the highest recovery of lung injury was in the preterm-UC-Muse group compared with the other 3 groups on day 14.

### Cardiorespiratory assessment

**Fig. 3:**
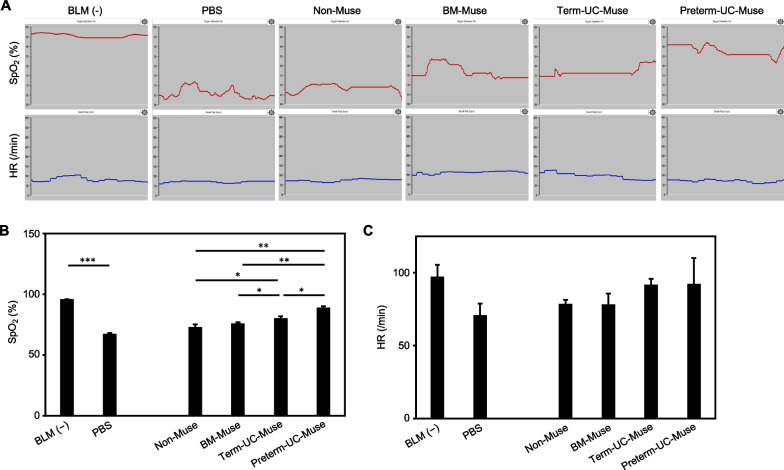
Pulmonary function measurements. **A** SpO_2_ and heart rate (HR) were recorded at day 9. Representative 30-s records for each group are presented. **B** SpO_2_ was measured for 60 min at day 9 (n = 3 for each group). *p < 0.05, **p < 0.01, and ***p < 0.001. **C** HR was measured for 60 min at day 9 (n = 3 for each group)

We next performed a cardiorespiratory assessment with a mouse/rat pulse oximeter on day 9 (Fig. 1A). SpO_2_ and heart rate were continuously recorded through a neck collar sensor fitted on freely moving awake rats. The basal SpO_2_ in the BLM(−) intact group (95.9 ± 0.1%) was significantly higher than that in the PBS group (67.4 ± 0.7%, *p* < 0.001), suggesting that BLM administration worsened lung function (Fig. 3A, B). Although the difference in SpO_2_ between the BM-Muse and non-Muse groups was not statistically significant, the preterm-UC-Muse group exhibited the highest recovery in SpO_2_ compared with the term-UC-Muse (*p* < 0.05), BM-Muse (*p* < 0.01), and non-Muse groups (*p* < 0.01; Fig. 3B). The term-UC-Muse group also had a higher SpO_2_ compared with the BM-Muse and non-Muse groups (both with *p* < 0.05).

In contrast to Sp0_2_, the change in the basal heart rate was less consistent: BLM(−) intact (97.3 ± 8.1/min), PBS (70.9 ± 7.9/min), non-Muse (78.7 ± 2.7/min), BM-Muse (78.2 ± 7.4/min), term-UC-Muse (92.0 ± 3.8/min), and the preterm-UC-Muse (92.4 ± 17.6/min) groups, with no statistically significant differences among them (Fig. 3C).

### Histopathologic analysis

**Fig. 4:**
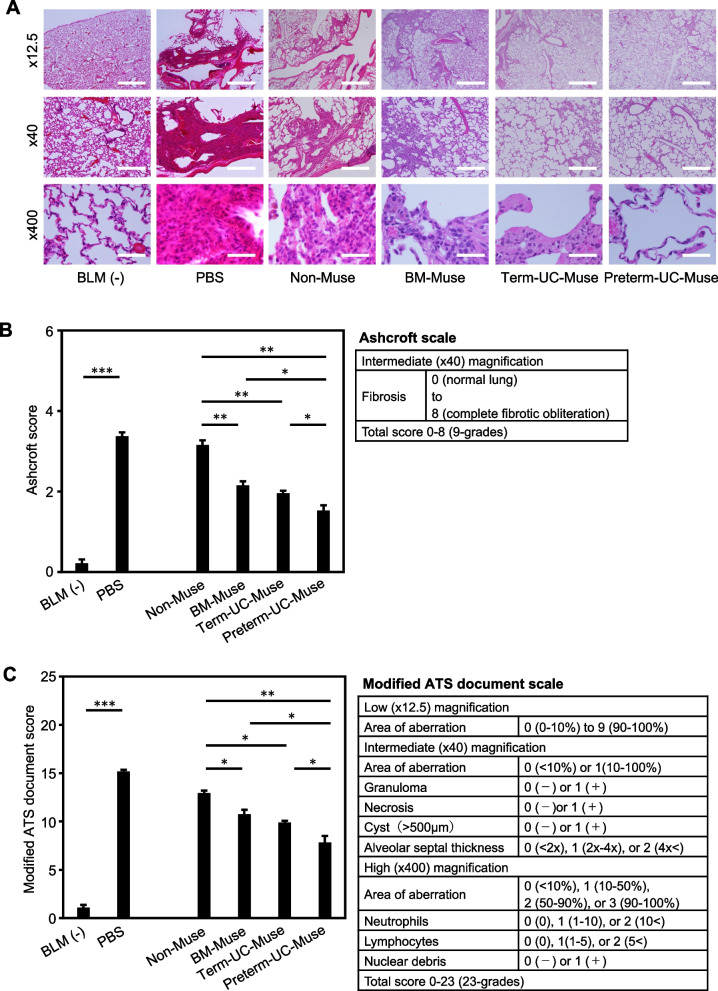
Histopathologic assessment. **A** Representative H&E images of the lung with low (× 12.5), intermediate (× 40), and high (× 400) magnifications. Scale bars; × 12.5 = 2 mm, × 40 = 500 µm, × 400 = 50 µm. **B** Lung fibrosis assessment. H&E sections (n = 5 for each group) were scored according to the Ashcroft scale. *p < 0.05, **p < 0.01, and ***p < 0.001. **C** Lung inflammation and fibrosis assessments. H&E sections (n = 5 for each group) were scored according to the modified ATS document scale. *p < 0.05, **p < 0.01, and ***p < 0.001

Histopathology of the lung was examined on day 21 (Fig. 1A). As shown in Figs. 4A and S1, the structural changes were clear in the PBS group compared with the BLM(−) intact group. Because BLM induces an acute inflammatory response that leads to a fibrogenic response in the early phase and fibrosis in the later phase, we used 2 scales to quantify the effect of cell treatment on lung histopathology.

The Ashcroft scale evaluates fibrosis and is the most widely used lung injury scale in animals; it assigns 9-step scores with 0 corresponding to normal lung and 8 corresponding to complete fibrotic obliteration of the lung [47, 48]. The Ashcroft scale score of the BLM(−) intact group was 0.22 ± 0.10, and that of the PBS group was 3.37 ± 0.10 (*p* < 0.001). The Muse cell group had a lower Ashcroft scale score than the PBS group; although the difference between the BM-Muse and term UC-Muse groups was not statistically significant, the preterm UC-Muse group (1.53 ± 0.13) had a lower score than the term UC-Muse (1.96 ± 0.06; *p* < 0.05), BM-Muse (2.15 ± 0.10; *p* < 0.05), and non-Muse groups (3.15 ± 0.12; *p* < 0.01; Fig. 4B).

As the Ashcroft scale evaluates only fibrosis, we also used the modified ATS document scale to evaluate inflammation and fibrosis degrees in the lung; it assigns 25-step scores with 0 corresponding to healthy lung and 24 to complete aberration [49, 50]. The BLM(−) intact group scored 1.07 ± 0.30, and the PBS group 15.18 ± 0.17 (*p* < 0.001). Consistent with the Ashcroft scale, the preterm UC-Muse group (7.84 ± 0.65) exhibited the lowest score compared with the term UC-Muse (9.91 ± 0.15; *p* < 0.05), BM-Muse (10.75 ± 0.46; *p* < 0.05), and non-Muse groups (12.91 ± 0.28; *p* < 0.01). Among the 3 groups, the term-UC-Muse, BM-Muse, and non-Muse groups, each was significantly different from the others except between the term-UC-Muse and BM-Muse groups (Fig. 4C).

### Engraftment of infused cells in BLM-induced lung injury model

**Fig. 5:**
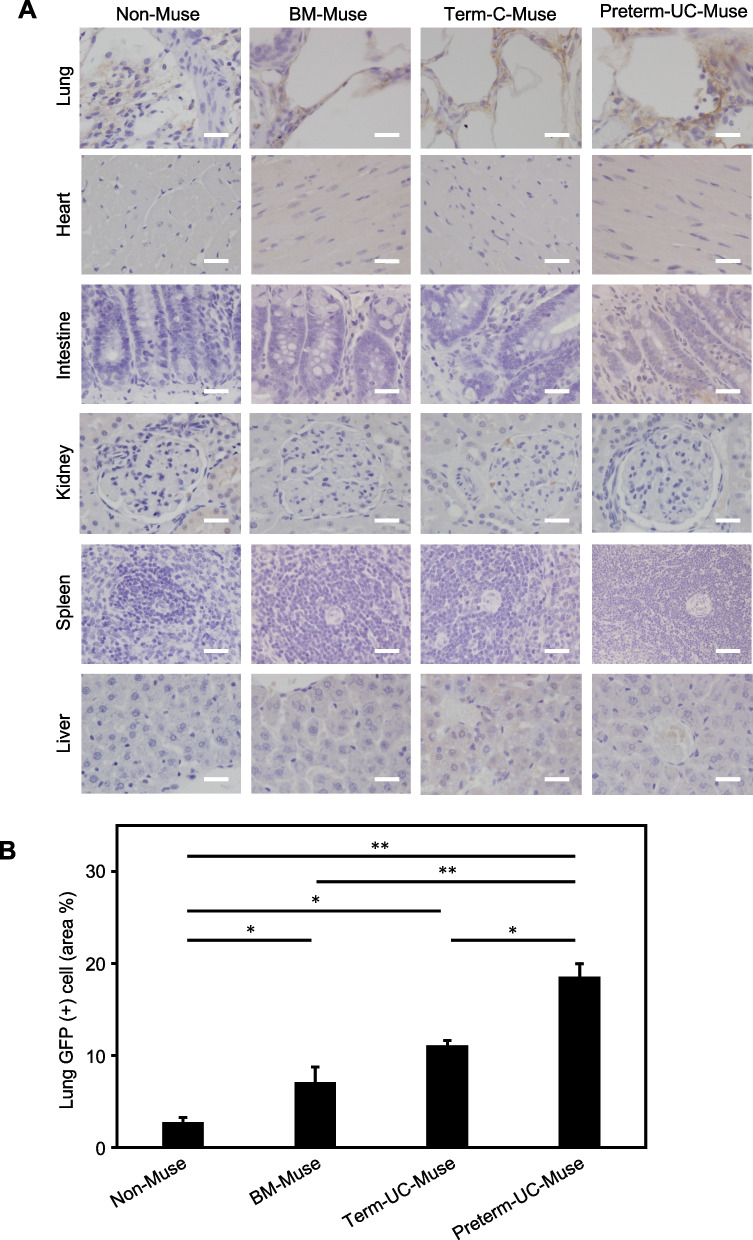
Engraftment of infused cells in the lung. Cells were introduced with GFP in all the groups. **A** Representative images of anti-GFP immunostaining in the lung, heart, intestine, kidney, spleen, and liver in the non-Muse, BM-Muse, Term-UC-Muse and Preterm-UC-Muse groups. Scale bars = 50 µm. **B** Quantification of GFP-positive cells detected in the lung (n = 3 for each group). Area % was defined as (GFP(+) cell area/total lung cell area) × 100. *p < 0.05 and **p < 0.01

Engraftment of GFP-labeled infused cells was further assessed in the lung, heart, intestine, kidney, spleen, and liver using anti-GFP immunostaining at day 21. GFP signal was detected in the lung, whereas it was rarely detected in other tissues in the preterm UC-Muse, term UC-Muse, BM-Muse, and non-Muse groups (Figs. 5A and S2). The percent of GFP-positive cell area to the total lung cell area was quantitated; the preterm UC-Muse group (18.54 ± 1.43%) had the highest value compared with the term UC-Muse (11.08 ± 0.56%; *p* < 0.05), BM-Muse (7.11 ± 1.62%; *p* < 0.01), and non-Muse (2.77 ± 0.49%; *p* < 0.01) groups (Fig. 5B). While the percent of GFP-positive cell area was not significantly different between the term UC-Muse and BM-Muse groups, the term UC-Muse and BM-Muse groups had a higher amount of engraftment compared with the non-Muse groups (*p* < 0.05; Fig. 5B).

### Immunohistochemical analysis for marker expression

We examined the marker expression of GFP-labeled cells engrafted in the lung at 21 days in the preterm-UC-Muse, term-UC-Muse, BM-Muse, and non-Muse groups. In the non-Muse group, not only was the number of GFP(+) cells the smallest, the expression of type I alveolar epithelial cell marker podoplanin, type II alveolar epithelial cell marker proSP-C, and vascular endothelial cell marker CD31 in those GFP(+) cells was hardly detectable (data not shown) [58]. Therefore, this group was omitted from the following experiment.

**Fig. 6:**
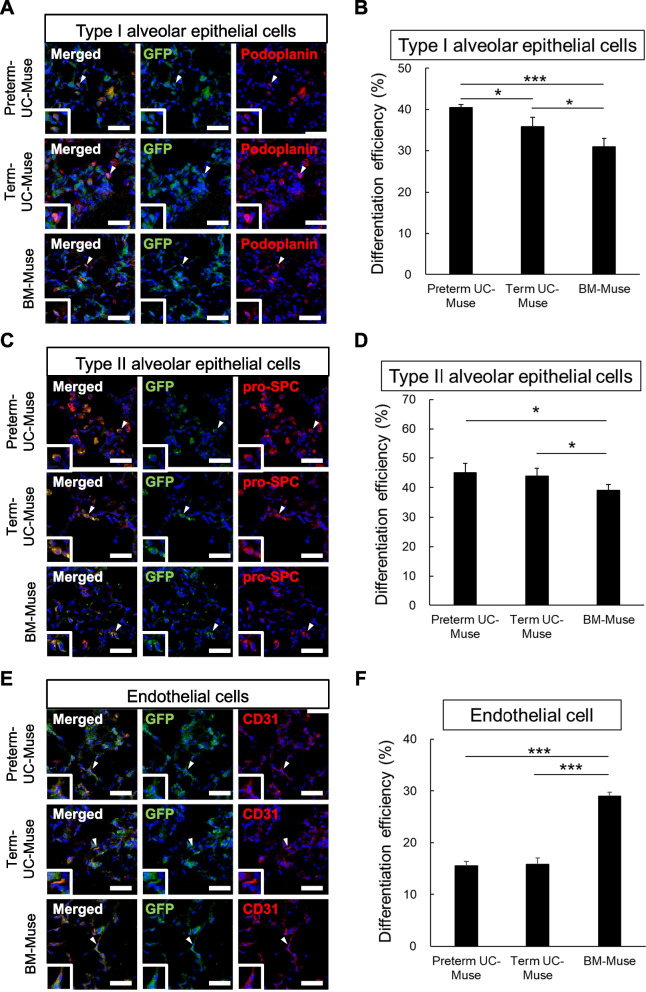
Marker expression of GFP-positive Muse cells in the lung. **A** Immunofluorescence for podoplanin (red), GFP (green), and DAPI (blue) in the preterm-UC-Muse, term-UC-Muse, and BM-Muse group. **B** The percent of podoplanin to the total GFP-positive cells. **C** Immunofluorescence for pro-SPC (red), GFP (green), and DAPI (blue) in the preterm-UC-Muse, term-UC-Muse, and BM-Muse groups. **D** The percent of proSP-C( +) cells among GFP( +) cells. **E** Immunofluorescence for CD31 (red), GFP (green) and DAPI (blue) in the preterm-UC-Muse, term-UC-Muse and BM-Muse groups. **F** The percent of CD31 to the total GFP-positive population. Scale bars = 50 µm. *p < 0.05 and ***p < 0.001

In the preterm UC-Muse group, 40.5 ± 0.8% of GFP( +) cells expressed podoplanin. In contrast, the ratio was 35.9 ± 2.2% and 31.0 ± 2.2% in the term UC-Muse and BM-Muse groups, respectively (preterm-UC-Muse vs. term-UC-Muse: *p* < 0.05, preterm-UC-Muse vs. BM-Muse: *p* < 0.001, term-UC-Muse vs BM-Muse: *p* < 0.05), suggesting the highest ratio of podoplanin(+)/GFP(+) cells in the preterm-UC-Muse group (Fig. 6A, B).

The percent of proSP-C(+) cells among GFP(+) cells was higher in the preterm UC-Muse (45.1 ± 3.1%; *p* < 0.05) and term UC-Muse (43.9 ± 2.6%; *p* < 0.05) groups compared with the BM-Muse (35.8 ± 2.8%) group, whereas the difference was not statistically significant between the preterm-UC-Muse and term-UC-Muse groups (Fig. 6C, D).

Regarding vascular endothelial cell marker CD31, 15.5 ± 0.8% of GFP(+) cells in the preterm UC-Muse group were positive, comparable to that of the term UC-Muse group (15.8 ± 1.2%; Fig. 6E, F). Notably, CD31(+) cells in GFP(+) cells were the highest in the BM-Muse group (29.0 ± 0.8%), significantly higher than that in the preterm UC-Muse (*p* < 0.001) and term UC-Muse (*p* < 0.001) groups (Fig. 6E, F).

### Differential gene expression and pathway analysis

**Fig. 7:**
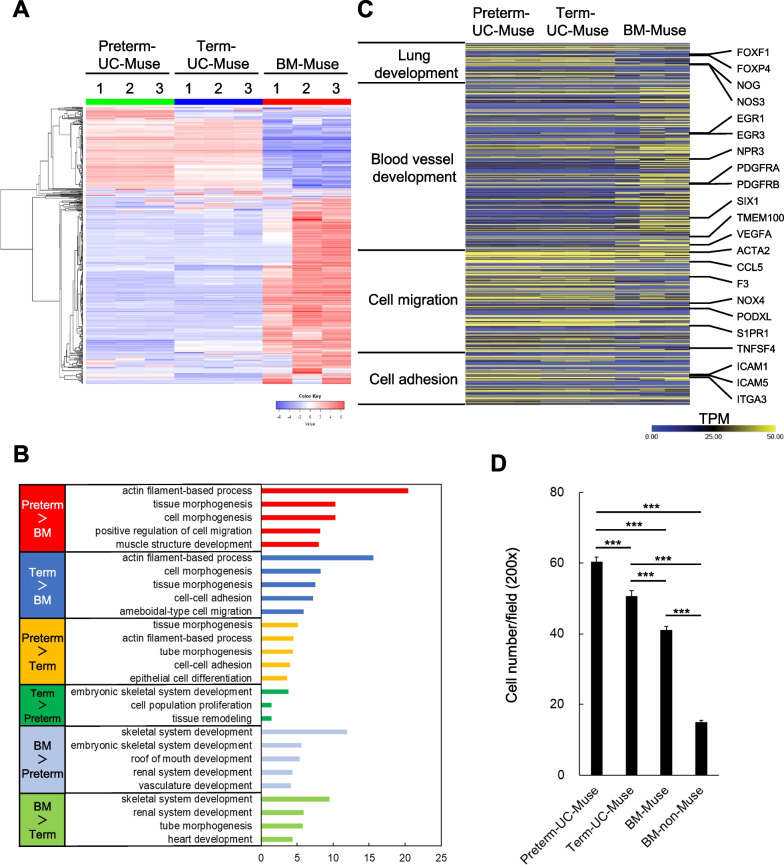
**A** Comparison of preterm-UC-Muse, term-UC-Muse, and BM-Muse cells in RNA-seq. Dendrogram and unsupervised hierarchical clustering heatmap of preterm-UC-Muse (1, 2, 3), term-UC-Muse (1, 2, 3), and BM-Muse cells (1, 2, 3). Degree of similarity between gene expression profiles is represented by the vertical distances on the dendrogram. The highest degree of correlation is represented by short vertical distances. **B** Gene ontology analysis: Upregulated genes in preterm (red)- and term (blue)-UC-Muse cells compared to BM-Muse cells are listed. Upregulated genes in preterm-UC-Muse (orange) and BM-Muse (light green) compared to term-UC-Muse cells are listed. Upregulated genes in term-UC-Muse (green) and BM-Muse (light blue) cells compared to preterm-UC-Muse cells are listed. **C** Heatmap of genes relevant to lung development, blood vessel development, cell migration, and cell adhesion. **D** Evaluation of the migratory ability of preterm-UC-Muse, term-UC-Muse, BM-Muse, and BM-non-Muse cells to the damaged lung tissue. The number of migrated cells was counted under 20 × objectives in 4 random fields for each sample, and the mean of 3 samples was used for comparison. ***p < 0.001

Three replicates of cultured preterm-UC-Muse, term-UC-Muse, and BM-Muse cells were subjected to RNA-seq. Genes with more than twofold differential expression were extracted for group comparisons, and pathway and process enrichment analyses were conducted. Genes with more than fourfold higher expression in BM-Muse than preterm- and term-UC-Muse cells were also identified (Fig. 7A).

Genes with more than twofold higher expression in preterm- and term-Muse cells than in BM-Muse cells were related to cell migration (actin filament-based process in both preterm- and term-UC-Muse cells, positive regulation of cell migration in preterm-UC-Muse cells, and ameboid-type cell migration in term-UC-Muse cells), and differentiation (tissue morphogenesis and cell morphogenesis in both preterm- and term-UC-Muse cells, cell–cell adhesion in term-UC-Muse cells; Fig. 7B). Genes with more than a twofold higher expression in preterm-UC-Muse cells compared with term-UC-Muse cells were also associated with vascular and epithelial differentiation (tissue morphogenesis, tube morphogenesis, epithelial cell differentiation), and migration (actin filament-based process, cell–cell adhesion). On the other hand, genes with more than a twofold higher expression in term UC-Muse cells compared with preterm UC-Muse cells were related to embryonic skeletal system development, cell population proliferation, and tissue remodeling.

Genes with more than a fourfold higher expression in BM-Muse cells compared with preterm- and term-UC-Muse cells were related to skeletal system development, renal system development, vascular differentiation (vasculature development when compared with preterm-UC-Muse cells, and tube morphogenesis when compared with term-UC-Muse cells), mouth development (preterm-UC-Muse cells), and heart development (term-UC-Muse cells; Fig. 7B).

Furthermore, gene expression related to lung- and blood vessel development, cell migration, and cell adhesion was compared. Both preterm- and term-UC-Muse cells exhibited higher expression of genes associated with lung development (e.g., forkhead box F1, forkhead box P4) [59], cell migration (e.g., coagulation factor III, S1P receptor 1) [26, 60], and cell adhesion (e.g., intercellular adhesion molecules 1 and 5) [61], compared with BM-Muse cells (Fig. 7C). On the other hand, BM-Muse cells exhibited high expression of genes related to blood vessel development, such as early growth response 3 and vascular endothelial growth factor A [62], than preterm- and term-UC-Muse cells (Fig. 7C).

### In vitro migration assay

In various animal models of injury, Muse cells are reported to selectively migrate to the damaged site via the S1P and S1PR2 axis [26]. The in vitro migratory ability of preterm-UC-Muse, term-UC-Muse, BM-Muse, and BM-non-Muse cells to the damaged lung tissue specimen obtained from intact Wistar rats was examined in vitro as described in the Methods section. Preterm-UC-Muse cells (60.2 ± 1.5 cells/field), term-UC-Muse cells (50.6 ± 1.6 cells/field), and BM-Muse cells (40.9 ± 1.2 cells/field) exhibited significantly higher migration compared with BM-non-Muse cells (all with *p* < 0.001; Fig. 7D). In addition, preterm- and term-UC-Muse cells exhibited higher migration compared with BM-Muse cells (both with *p* < 0.001), and pre-term-UC-Muse cells exhibited higher migration than term-UC-Muse cells (*p* < 0.001; Fig. 7D).

## Discussion

BLM-induced lung injury shows sequential events of an acute inflammatory response, fibrogenic changes, extracellular matrix deposition, and alterations of the smooth muscle and microvasculature. As human lung diseases are basically characterized by a combination of inflammation and fibrosis, BLM-induced lung injury is widely used as an animal model for the preterm infant-related lung complication BPD [5, 6] as well as age-related lung diseases such as IPF and COPD [4, 7]. While exhaustion and/or dysfunction of resident lung stem cells are found in preterm infants with BPD [10, 11] and aged patients with COPD [63], the preventive or therapeutic effects of MSCs are limited due to their inability to engraft into the injured lung and differentiate into lung components.

Muse cells have several unique beneficial characteristics; surgical treatment is not necessary but noninvasive intravenous administration is fully effective for delivering Muse cells to the target injury tissue due to sensing S1P produced by damaged/apoptotic cells [26]; no need for gene transfer or differentiation induction because Muse cells are already pluripotent-like and can spontaneously differentiate into the target cell type to replace damaged/apoptotic cells by phagocytosis-induced differentiation mechanism in the homed injured tissue [21, 23]; and no need for HLA-matching and immunosuppression, partly due to the expression of HLA-G, relevant to immune tolerance in the placenta [26, 30]. These advantageous properties have been demonstrated in a variety of animal models [26, 64–68], and the safety and efficacy of clinical grade BM-derived Muse cells have been reported for several diseases such as acute myocardial infarction, subacute stroke, epidermolysis bullosa, and amyotrophic lateral sclerosis [31–34].

While SSEA-3-positive Muse cells were harvested from human UC tissue in a previous report [38], differences in the basic characteristics and therapeutic effects between preterm- and term-UC-Muse cells have not been evaluated until the present study. Here, we intravenously administered human Muse cells without immunosuppressive agents into a rat BLM-induced lung injury model and compared the therapeutic efficacy between preterm- and term-UC-Muse cells, and between preterm/term-UC-Muse cells and BM-Muse cells (as standard Muse cells). MSCs have been extensively tested in clinical trials [69], and Muse cells comprise several percent of MSCs [21]. To critically assess the therapeutic efficacy of MSCs, we used non-Muse MSCs, i.e., MSCs that represent 97–99% of the total population, excluding Muse cells, for comparison.

The findings of the present study suggested that preterm-/term-UC-Muse cells were superior to BM-Muse and non-Muse cells for the recovery of weight loss, serum SP-D levels, SpO_2_, and the Ashcroft and modified ATS document scores. Preterm-/term-UC-Muse cells also exhibited significantly better homing ability to the injured lung and significantly higher expression of type 1- and type 2-alveolar cell markers compared with BM-Muse and non-Muse cells. Comparing between preterm- and term-UC-Muse cells, the preterm UC-Muse cells exhibited significantly superior recovery in the serum SP-D level, SpO_2_, and the Ashcroft and modified ATS document scores, and superior homing to the lung and expression of type 1-alveolar cell marker than the term UC-Muse cells.

The basis for these differences among preterm- and term-UC-Muse cells and BM-Muse cells was examined in terms of their gene expression patterns and in vitro migration to injured lung tissues. The expression of genes related to cell migration, cell adhesion, and differentiation, particularly relevant to lung differentiation, was higher in preterm-/term-UC Muse cells than in BM-Muse cells, while on the other hand, BM-Muse cells showed higher expression levels of genes related to skeletal, renal, vascular, and heart differentiation factors than preterm-/term-UC-Muse cells. The higher proportion of cells positive for type 1- and type 2-alveolar cells among the cells homed to the lung was suggested to be related to the higher expression of forkhead box F1 and forkhead box P4, which are relevant to lung differentiation, in preterm-/term-UC-Muse cells than in BM-Muse cells [59]. The expression of factors related to vascular and epithelial differentiation, as well as migration, was higher in preterm-UC-Muse cells than in term-UC-Muse cells.

In the in vitro migration assay, preterm-/term-UC-Muse cells showed higher migration ability than BM-Muse cells to the lung slice tissue, and notably, preterm-UC-Muse cells showed even higher migration than term-UC-Muse cells, consistent with the differential gene expression results mentioned above. Taken together, the present results suggest that human preterm-UC-Muse cells would be expected to have therapeutic effects superior to those of term-UC-Muse and BM-Muse cells for lung diseases such as BPD, IPF, and COPD.

Preterm-UC-Muse cells have several advantages over other Muse cells. First, the UC can be obtained non-invasively during newborn delivery, different from BM-Muse cells that require invasive sampling from the donor BM. Second, as UC-MSCs have higher proliferation capacity than BM-MSCs, as reported previously [70, 71], UC-Muse cells might be easier to harvest on a clinical scale than BM-Muse cells. In terms of gestational age of the UC, preterm UC yields more MSCs and possibly more Muse cells than the term UC, because preterm-UC-MSCs exhibit more vigorous proliferation and higher differentiation capacity than term-UC-MSCs [38–40]. Although UC blood is included in the UC, the recommended delayed cord clamping for preterm infants makes UC blood sampling difficult [72]. Therefore, preterm UC-MSCs might be a better source for collecting Muse cells than the UC blood from preterm infants. On these bases, preterm-UC-Muse cells are expected to be an ideal source for stem cell therapy for treating human patients with BPD, IPF, and COPD.

However, there are some limitations in the present study. One limitation is the dose of human Muse cells. Because human Muse cells exhibited the dose-dependent therapeutic effects on a mouse stroke model [73], their effects on a rat lung injury model are expected to be dose dependent. Accordingly, the Muse cell dose (1 × 10^5^ cells) in the rat BLM-induced lung injury model was estimated from the Muse cell dose used in the rat ischemia–reperfusion-induced lung injury model [43]. Further study is needed to determine the optimum Muse cell dose for the rat BLM-induced lung injury model. The other limitation is the use of rat BLM-induced lung injury model. Although the BLM-induced lung injury model is considered the gold standard for inducing lung injury, it may insufficiently mimic the onset and progressive nature of human lung disease. To benefit human patients with BPD, IPF, and COPD, well-designed clinical trials are required.

## Conclusions

Clinical trials for some human diseases have suggested the safety and therapeutic efficacy of intravenously injected human leukocyte antigen-mismatched allogenic Muse cells from adult BM without immunosuppressant. However, there are no study comparing the therapeutic efficacy of human Muse cells from adult BM and other sources. Our present results demonstrate that preterm UC-Muse cells deliver more efficient therapeutic effects than term UC- and BM-Muse cells for treating BLM-induced lung injury in a rat model.

### Supplementary Information

Supplementary Material.

## Data Availability

The datasets discussed in the present study have been deposited in NCBI's Gene Expression Omnibus (GEO) and are accessible through GEO Series accession number GSE263992 (https://www.ncbi.nlm.nih.gov/geo/query/acc.cgi?acc=GSE263992).
