# Supplementary material for: Human Muse cells isolated from preterm- and term-umbilical cord delivered therapeutic effects in rat bleomycin-induced lung injury model without immunosuppressant
Source: Stem Cell Res Ther. 2024 May 22;15:147. doi: 10.1186/s13287-024-03763-8 (PMC11110192; doi:10.1186/s13287-024-03763-8)
Supplement: Supplementary file 1 — Supplementary Material. [file 13287_2024_3763_MOESM1_ESM.pdf]

Supplementary Table S1

Human UC samples

#	Preterm or Term	Sex	Gestational age
1	Preterm	Male	23 week 0 day
2	Preterm	Female	23 week 6 day
3	Preterm	Female	23 week 1 day
4	Term	Female	38 week 1 day
5	Term	Female	38 week 3 day
6	Term	Female	38 week 2 day

Supplementary Figure S1

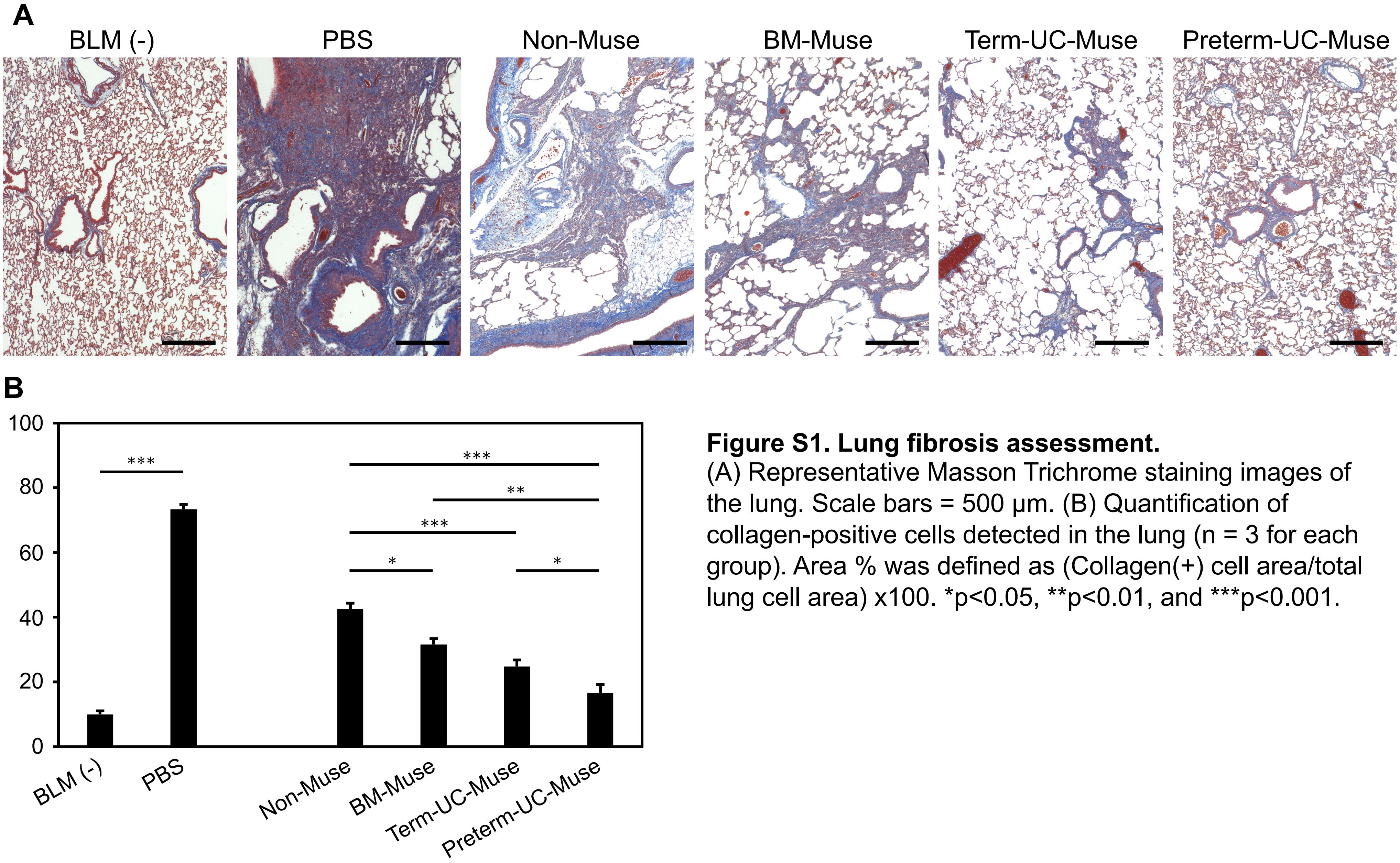

Figure S1. Lung fibrosis assessment.
(A) Representative Masson Trichrome staining images of the lung. Scale bars = 500 μm. (B) Quantification of collagen-positive cells detected in the lung (n = 3 for each group). Area % was defined as (Collagen(+) cell area/total lung cell area) x100. *p<0.05, **p<0.01, and ***p<0.001.

Supplementary Figure S2

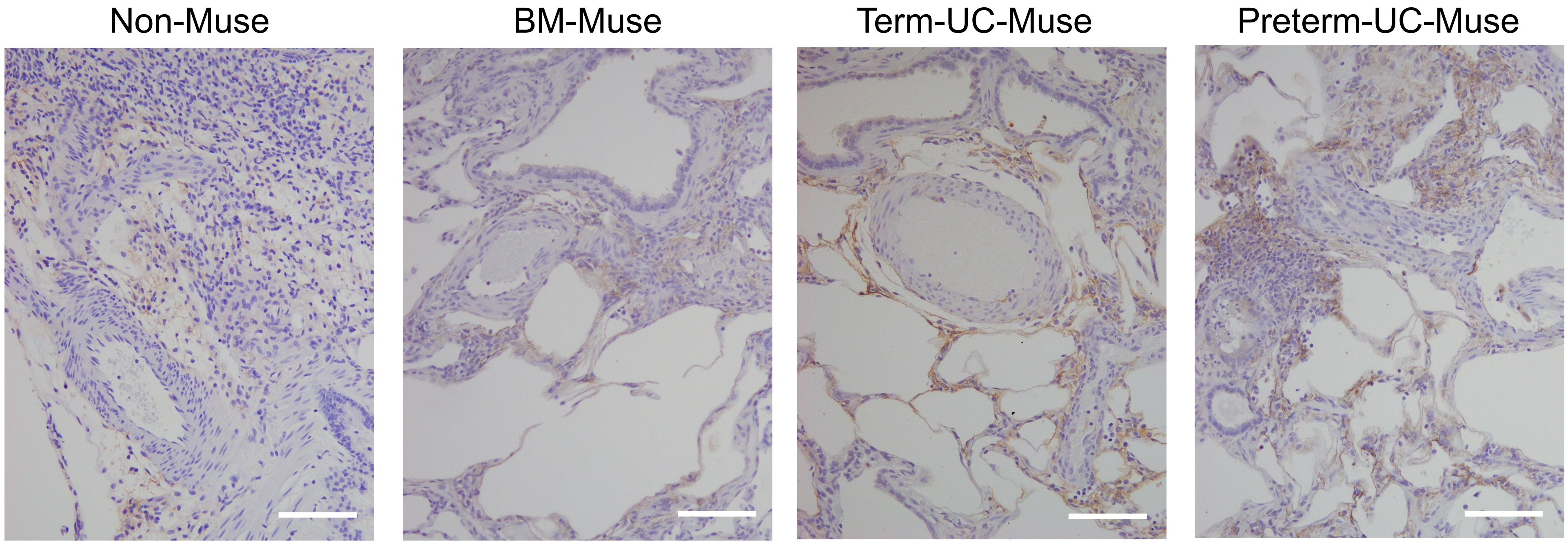

Figure S2. Engraftment of infused cells in the lung.
Cells were introduced with GFP in all the groups. Low magnification images of anti-GFP immunostaining of the lung in the non-Muse, BM-Muse, Term-UC-Muse and Preterm-UC-Muse groups. Scale bars = 100 μm.
